# Supplementary figures and images for: Biomolecules involved in the metabolism of Escherichia coli affected by photodynamics: A calorimetry study
Source: PLoS One. 2026 Apr 21;21(4):e0347474. doi: 10.1371/journal.pone.0347474 (PMC13098931; doi:10.1371/journal.pone.0347474)

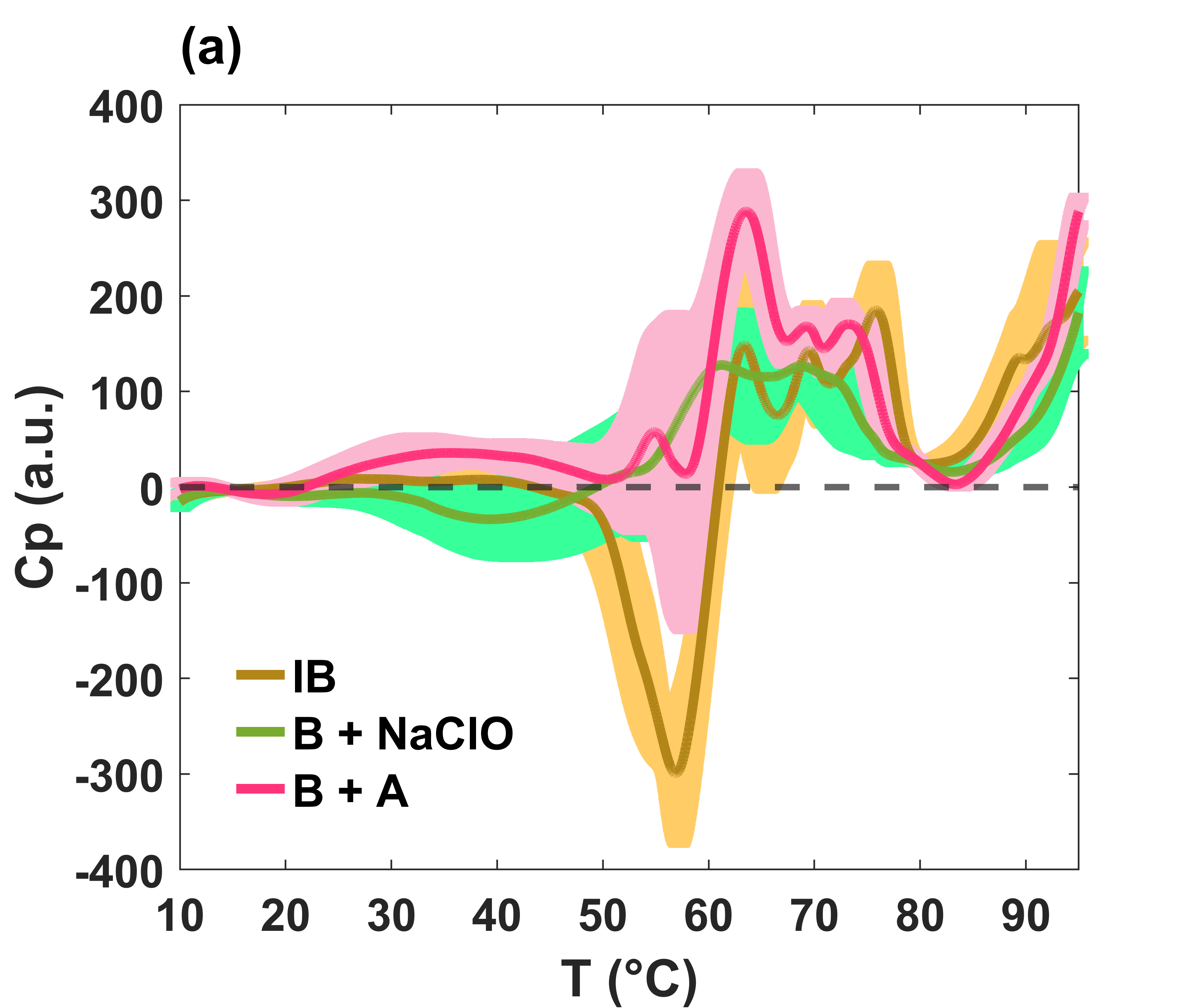

Supplement: S1 Fig — Bacteria with sodium hypochlorite (B + NaClO) and bacteria with ampicillin (B + A). There is a significant reduction in the metabolic valley in bacteria treated with NaClO and its disappearance in ampicillin cultures. Three measurements of each sample were performed. The average is displayed in a thin line, while the shadow band corresponds to the standard deviation. (JPG) [file pone.0347474.s001.jpg]

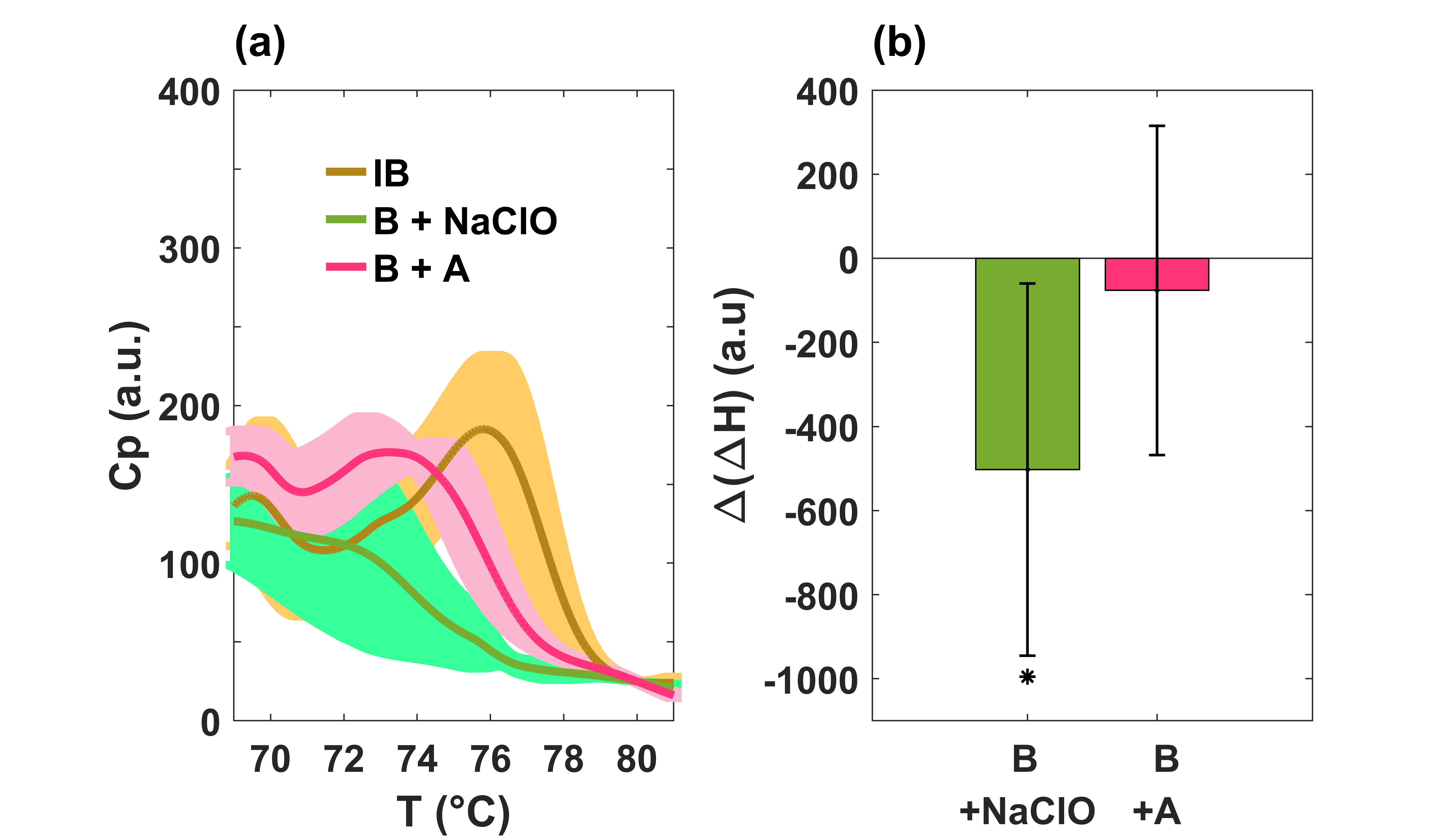

Supplement: S2 Fig — (a) Calorimetry profiles of the protein region for bacteria treated with sodium hypochlorite (B + NaClO) and bacteria with antibiotic (B + A) compared to control (IB). (b) Enthalphy change differences with respect to the control sample showed a reduction in significance only for the first case. Three measurements of each sample were performed. The average is displayed in a thin line, while the shadow band corresponds to the standard deviation. (JPG) [file pone.0347474.s002.jpg]

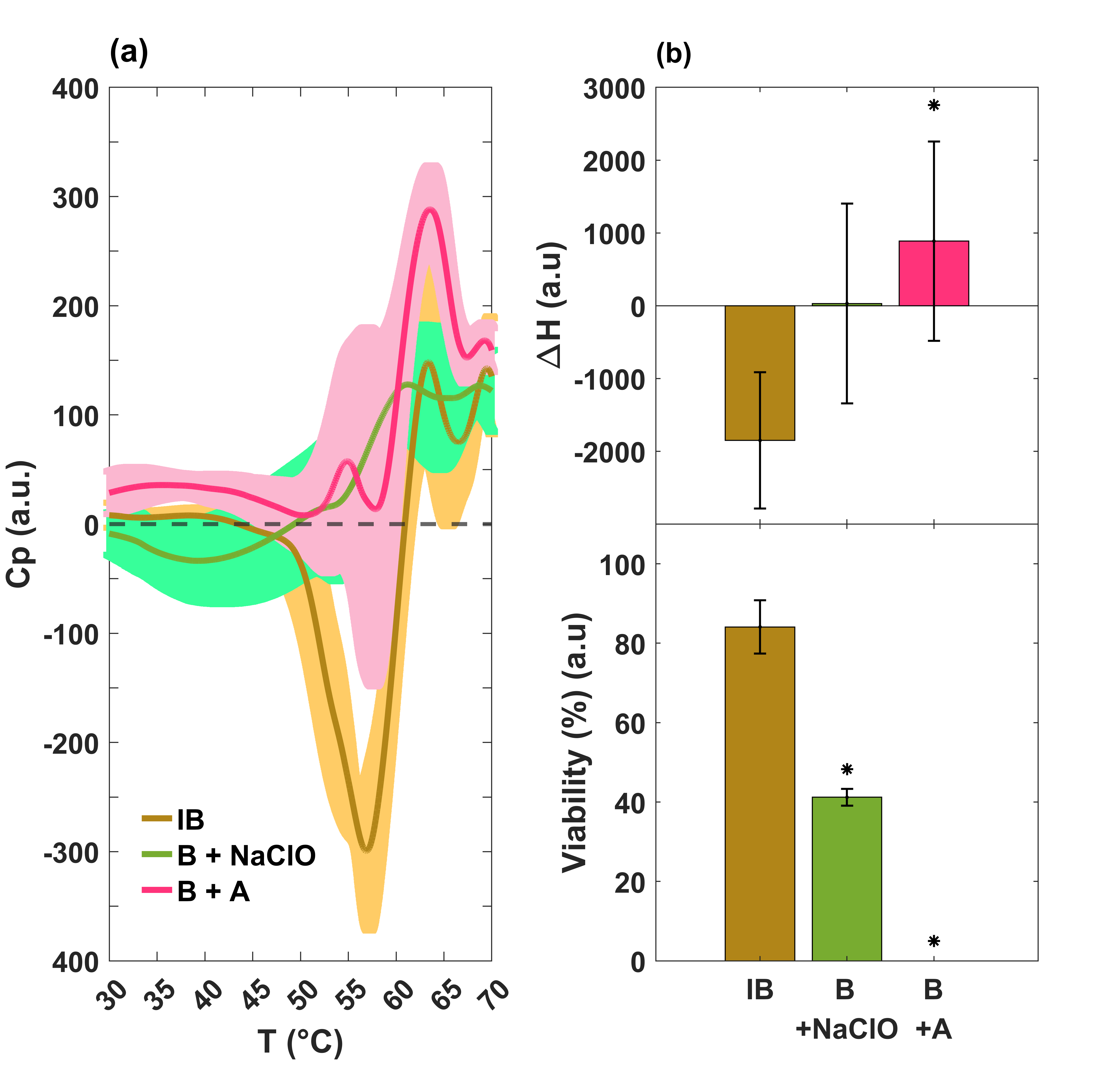

Supplement: S3 Fig — (a) Amplification of the exothermic valley. There is a significant reduction in the metabolic valley in bacteria treated with NaClO (B + NaClO) and its disappearance in cultures with ampicillin (B + A). (b) Comparison between the enthalpy change differences and viability. The treatments exhibit a similar behavior. Three measurements of each sample were performed. (JPG) [file pone.0347474.s003.jpg]
